# Promoting Global Health Knowledge and Cultural Competence of Swedish and Somali Nursing Students Through Collaborative Virtual Seminars: A Qualitative Descriptive Study

**DOI:** 10.1177/10436596241271088

**Published:** 2024-08-16

**Authors:** Olivia M. Örtlund, Inger Andersson, Fatumo Osman

**Affiliations:** 1 Dalarna University, Falun, Sweden

**Keywords:** cultural competence, global health, qualitative, nursing education, internationalization

## Abstract

**Introduction:**

Engaging nursing students in transdisciplinary learning and collaborative activities will prepare them for future roles in promoting global health knowledge and cultural competence. The purpose of this study was to describe undergraduate nursing students’ experiences of participating in collaborative virtual seminars intended to promote global health knowledge and increase cultural competence between a university in Sweden and a university in Somaliland.

**Methods:**

A qualitative descriptive study using semi-structured individual interviews and focus group discussions was conducted. Notably, 27 nursing students who participated in a collaborative virtual seminar were included. Data were analyzed using inductive qualitative content analysis.

**Results:**

Nursing students reported having gained profound comprehension of and a broadened perspective on global health, cultural awareness, and curiosity crucial for their roles as future global nurses.

**Discussion:**

Fostering global health knowledge and cultural competence among nursing students through collaborative virtual seminars is advantageous as it enhances their cultural competence in nursing.

## Introduction

As understanding global health requires significant societal and pedagogical transformations, universities are responsible for engaging students in transdisciplinary learning and involving them in collaborative international educational activities (Collins et al., 2018; El-Jardali et al., 2018). Virtual collaborative international learning has the potential to transform nursing students into international thinkers and leaders. This approach offers feasible, meaningful, and cost-effective solutions, thereby enriching cultural competence and global understanding of health through virtual knowledge exchange (Bragadóttir & Potter, 2019; Jung et al., 2022; Kiegaldie et al., 2022; Potter & Bragadóttir, 2020). This study describes how a collaborative virtual seminar for nursing students at two universities promoted global health knowledge and cultural competence.

## Background

Global health is an area of study, research, and practice that prioritizes improving health and equality in health for all people worldwide (Wilson et al., 2016). Global nursing is an evidence-based nursing process that promotes sustainable planetary health and equity for all people (Rosa et al., 2019; Wilson et al., 2016). Nurses can affect global health through education (Fields et al., 2021).

To promote global health, universities have been urged to be actively involved in collaborative international learning (Mallow et al., 2020; United Nations [UN], 2015). Upvall and Luzincourt (2019) stated that using global health as a platform for education, creating transformational changes, and raising awareness of national and global health challenges have the potential to empower future nurses to make significant impacts in the field of health. Nurses, the largest body of health care staff (Mallow et al., 2020), should engage in ethical practices and show respect for human dignity, human rights, and cultural diversity. This will enable them to offer safe, adaptive, and meaningful patient care (McFarland & Wehbe-Alamah, 2018; Sanford et al., 2021; Sharifi et al., 2019).

Cultural competence is a central aspect of most nursing programs and is considered a vital skill. Campinha-Bacote (2002) identified cultural competence as a process whereby nurses develop the skills, desire, integrity, sensitivity, knowledge, and cultural awareness that empower them to work in transcultural situations. Campinha-Bacote has also developed the concept “cultural competemility,” which is a synergistic relationship between cultural competence and cultural humility (Campinha-Bacote, 2018). Nurses should maintain a position of cultural competemility as they engage in cultural encounters, obtain cultural knowledge, conduct culturally sensitive assessments, and become aware of their own biases (Campinha-Bacote, 2018). International student exchange programs enable nursing students’ personal and professional development (Antón-Solanas et al., 2021; Sharifi et al., 2019), cultural awareness, and understanding of fundamental behavior, attitudes, and beliefs beyond their own worldviews and comfort zones (Gower et al., 2017; Jansen et al., 2021).

However, with the development of technology, these benefits of international learning do not always require international student exchange. Universities employ collaborative virtual international learning to enhance nursing students’ understanding of global health, cultural competence (Fadaeinia et al., 2022; Kunaviktikul & Turale, 2020), and the practice of internationalization at home (laH; non-outbound student mobility/not studying abroad).

laH is defined as “any internationally related activity with the exception of outbound student and staff mobility” (Crowther et al., 2000). It enables students to be involved in purposeful international and intercultural activities both at home and in other countries (Chen et al., 2018; Hyett et al., 2019; Jansen et al., 2021; Kiegaldie et al., 2022; King et al., 2021; Limoges et al., 2019; Potter & Bragadóttir, 2020; Wihlborg et al., 2018). The benefits of collaborative virtual international learning include new learning experiences in an asynchronous classroom, exposure to diverse views, promotion of novel ideas, and the opportunity to learn different health care delivery models and systems (Bragadóttir & Potter, 2019; Jung et al., 2022; Kiegaldie et al., 2022; Potter & Bragadóttir, 2020). Fadaeinia et al. (2022) found that online cultural care training programs can effectively promote nursing students’ cultural competence and self-efficacy.

Despite increased awareness of universities’ central role in implementing global health and cultural competence in nursing curricula through collaborative virtual international learning, further collaboration between the Global North and Global South is required (Bragadóttir & Potter, 2019; El-Jardali et al., 2018; Fadaeinia et al., 2022; Kunaviktikul & Turale, 2020; Mallow et al., 2020; McFarland & Wehbe-Alamah, 2018; Potter & Bragadóttir, 2020; Sanford et al., 2021). The purpose of this study was to describe undergraduate nursing students’ experiences of participating in collaborative virtual seminars intended to promote global health knowledge and increase cultural competence between a university in Sweden and a university in Somaliland.

## Method

### Design

A qualitative descriptive design employing individual interviews and focus group discussions was used. The consolidated criteria for reporting qualitative research (COREQ) were used to report the findings of this study and enhance the transparency and reproducibility of the study (Tong et al., 2007).

### Setting

This study was part of a collaborative project between two universities—one in Somaliland and one in Sweden—that began in 2013. The project’s primary goal was to introduce online education for nursing and midwifery students in Somaliland. This study examined one of the activities—a collaborative virtual seminar—conducted as part of the project. The seminar’s aim was to present and discuss global and national public health priorities and the nurse’s role in identifying sustainable solutions for global health and well-being. Students were asked to work in groups and select one area for in-depth study related to climate change and public health, including the related consequences for people’s physical and mental health, society, the economy, and the environment. They were also asked to discuss how nurses could promote health in relation to their selected area of interest. The seminar took place on Zoom and lasted for 4 hrs.

Students from the University of Hargeisa (UoH), Somaliland participated collectively from their university laboratory, a few students from Dalarna University (DU), Sweden joined from the campus laboratory, and others joined from their homes. The seminar presentations were organized within the students’ respective groups, and the seminar was attended by students from both universities. The course coordinators planned the seminar layout, discussion topics, and schedule for virtual technical support. Although the participants from the two universities were attending two different courses, both courses were related to social perspectives on health; for the collaborative virtual seminar, the students from both universities were given the same tasks and guidelines. Across a 2-year span (2018–2020), three collaborative virtual seminars with 58 undergraduate nursing students from UoH and DU were conducted.

### Participants

Purposeful convenience sampling was used to recruit participants for the study. The participants from DU were first-year undergraduate nursing students registered in a course entitled “Humanity and Health in a Societal Perspective.” The participants from UoH were third-year undergraduate nursing students enrolled in a community health nursing course. The students were informed about the collaborative virtual seminar, and their participation was requested by the first and last authors. They also received information indicating that they could participate in the collaborative virtual seminar without participating in the study.

In total, 58 nursing students were approached—24 from UoH and 34 from DU—all of whom participated in the three distinct collaborative virtual seminars. Among them, 27 (15 females, 12 males) agreed to participate in this study, with 12 from UoH and 15 from DU. The age range for UoH students was 20–44 years and that for DU students was 20–25 years.

### Data Collection

Data were collected for the period from 2018 to 2020, using semi-structured interviews conducted in four focus group discussions (*n* = 23), two individual interviews using video conferencing, and two individual interviews on the DU campus. The variety of interview formats was guided by participant availability. Initially, the participants were invited to focus group discussions. However, when they were unavailable, we explored options for conducting interviews either in person or via video conferencing. A predefined thematic interview guideline was used covering the following themes: experiences of participating in the collaborative virtual seminar, global health knowledge, and cultural awareness and competence (Table 1).

**Table 1.** Interview Guide.

Themes/topics	Questions
Participation in the collaborative virtual seminar	- What motivates you to participate in the collaborative virtual seminar? - What is your experience of taking part in collaborative virtual seminar? - Can this form of interaction with nursing students from other countries contribute to cultural awareness, cultural knowledge, or cultural ability? Why? Why not? How?
Global Health Knowledge	- What experiences in the global health knowledge does collaborative virtual seminar contribute or not contribute to? - What knowledge or insights have you gained from the seminars on global health?
Cultural awareness and competence	- How has collaborative virtual seminar contributed or not contributed to your cultural awareness? - In what way have you become aware of yourself and others after attending the collaborative virtual seminar? Can you give examples? - What cultural competence has collaborative virtual seminar contributed to? - In what ways have collaborative virtual seminar helped you increase your intercultural skills?

Campinha-Bacote’s (2002) framework for cultural competence inspired the interview questions related to cultural awareness and competence. However, we intended to obtain the informants’ perspectives and not rely heavily on the framework. Open-ended questions allowed the students to discuss their experiences. The interview guide was pilot-tested with the first focus group; since substantial changes were not made, we included the pilot focus group in the analysis. All interviews were digitally recorded with the participants’ consent. The focus group discussions and individual interviews were conducted separately for the students from each university, as we wanted to understand the respective experiences of each group. The interviews with the DU nursing students were conducted in Swedish, and those with the UoH nursing students were conducted in English. These languages align with those of the students’ respective university curriculum and language of study.

Each focus group comprised three to seven participating students. The focus group discussions were led by a moderator and observed by a notetaker, both of whom were involved in facilitating the seminar but were not responsible for grading the course. Both the moderator and observer are female and fluent in both Swedish and English. They are both nurses and experienced university lecturers with expertise in leading focus group discussions. This expertise enabled them to act confidently in intervening in or facilitating discussions among the participants (Krueger & Casey, 2014).

The last author, an associate professor, has conducted previous studies using focus group discussions. The moderator and observer clarified that they were not responsible for grading the course and that the participants had the option to withdraw at any time. Immediately after each focus group discussion, the moderator and the observer summarized their reflections on the discussions. Their notes and reflections were used to support the analysis of the results. The individual interviews were conducted by the first author and lasted 30–45 min. The focus group discussions were conducted by both authors and lasted 45–60 min. The interviews continued until no new information emerged.

### Analysis

The data were manually analyzed following the inductive qualitative content analysis method proposed in the work by Elo and Kyngäs (2008), which is used to systematically comprehend data to describe and explain a specific phenomenon. The interviews and focus group discussions were transcribed verbatim, and the analysis was conducted in the original language in which the interviews were conducted. The analysis began with the first and last authors reading the transcribed text individually to gain an overall understanding of it and to identify its fundamental implications. In the second stage, the units of meaning relevant to the study’s purpose were selected from the transcribed text. In the third stage, the first author focused on the manifest content, organized the data into codes, and grouped the coding data into categories and subcategories (Table 2).

**Table 2.** Analytical Process.

Manifest content	Code	Subcategory	Category
We learned about differences in climate change. What type of climate change, causes, how it affects people, and how it’s managed by the two countries (FGD 1, Student 2, Somali). It was interesting to know that the outcomes of climate change in our countries were different. The climate change causes flood and droughts”? but in Sweden, it courses fire and agricultural hazards. I also benefit from learning that climate change can affect and increase . . . communicable diseases like (FGD 2, Student 1, Somali). The benefit of having this type of student knowledge exchange through collaborative virtual seminar was that it allowed us to find out and get to know how other think and do things differently in order to reach the SDGs goals (FGD 2, Student 2, Swedish). We have come a long way, but still quite a lot of work needs to be done with the global climate change problems. I think through collaborative virtual seminar, we, nursing students, can contribute by sharing experiences and building ideologies that prevent and sustain from a global perspective (ID, Student 4, Swedish). We have learned how different climate change problems can be managed . . . and how we can manage as nurses. Because as a future nurse, you are supposed to work globally and not only in your country (FGD-1, Student 3, Somali). I remember it was during summer, and it was so hot, and every one of us Swedish students had water bottles in our hands, while they told us about their water shortage. You get a bad conscience when sitting there and with the advantage, but at the same time, you should not really be ashamed of it either . . . but we should be grateful for it and try to share what we have (FGD-3, Student 2, Swedish).	Learning from each other about differences and similarities in global health Learning about our similarities and differences Broaden the knowledge on global health issues Possibility for exchanging knowledge and building ideologies in sustaining global perspective Learning and becoming global nurses Reflection on own privilege and resources	Understanding differences in global health	Putting global health into perspective
It was so good reading and getting theoretical knowledge before doing the collaborative virtual seminar because then we could relate to and implement the theoretical knowledge in practice (FGD-3, Student 3, Swedish). It is different to read it in the books and gain understanding than to actually hear from people who live in it (FGD-1, Student 3, Swedish). I had an AHA! experience. I got an understanding in black and white of how it is. It is not just something I read about (ID, Student 1; Swedish).	Relating to, understanding theories, and putting it in praxis Better understanding of theories when received in praxis Gained a better understanding and confirmation of the learned theory	Understanding theories in depth	
During collaborative virtual seminar we got to reflect on the fact that we do have different people in our country and we meet them in our clinic and sometimes when we meet them, they don’t tell you everything because we have different culture . . . and participating in this seminar made us to be aware that we need to encounter them in a different way . . . We have to welcome them and be sensitive . . . culturally sensitive (FGD-1, Student 3, Somali). Yes, it’s more of the fact that a lot of people come here . . . Now we know that it is a completely different world we live in and to be able to meet them, we know a little of what they have with them from before. I have gained a little more understanding which is good to have so that one do not treat everyone the same . . . but that you can really think person-centered . . . (ID, Student 1, Swedish). . . . I reflect on . . . that one must ignore one’s own cultural aspects so that you do not care for a person in a way that they get offended in anyway (FGD-3, Student 2, Somali). It [collaborative virtual seminar] has significantly increased my awareness of other cultures. With the twinning you got a food for thought . . . (ID, Student 2, Swedish). . . . understanding that others have it in other ways . . . they have a completely different background they come from and a completely different culture and values (ID, Student 2, Swedish).	Being culturally sensitive in encountering with people with other cultural backgrounds Being aware of cultural differences and being sensitive to encountering people with different cultural backgrounds Reflecting on cultural aspects to give good patient care (Experiences from twining increase cultural awareness) Gaining awareness and sensitivity for others’ cultural backgrounds and values	Cultural awareness and transparency	Increasing cultural competence
For me . . . it probably makes me more responsive, humble, responsible, and better at taking others into account, as well as their cultural background. This humility will help us when we start working as nurses, I hope (ID, Student 4, Swedish). Yes . . . I have become grateful for everything I had . . . the conditions I have, I must be grateful for that. Yes I have to admit that . . . having more respect for others (ID, Student 1, Swedish). Meeting and discussing with the Somali students opened my eyes absolutely (ID, Student 3, Somali). What do I have with me here? That you might question yourself a little . . . it will probably be good if you might reflect a little on so I have thought about that a little (FGD, Student 7, Swedish). We were surprised how good they were in communicating in a completely different language (English). Many of us had prejudices regarding low-income countries, but from our experience from the collaborative virtual seminar (ID, Student 3, Swedish). It was interesting seeing that the Swedish nursing students were of different races. There were white students and black students, and we could see how well they interact with each other (FGD-2, Student 1, Somali).	Becoming humble, more responsible, and considerate of other cultures Become grateful and more respectful of others Self-growth and getting new perceptions Gaining self-reflection Having change of preconception Gaining new cultural perceptions on Swedish nursing students	Change in attitude and self-growth	

Thereafter, to maintain both credibility during the analysis and the validity of the results, the last author read the material analyzed up to that point and cross-checked it with the codes. The first and last authors re-examined the material, considering the various interviews, and agreed on categories and subcategories relevant to the study’s purpose. Descriptive quotations from the interviews were selected and placed in the text to strengthen and provide credibility to the analysis. Throughout the analysis, the authors critically reviewed, reflected on, and discussed the findings, consciously striving to avoid their own preconceptions.

### Ethical Considerations

Ethical approval for the study was requested from the Swedish Ethical Review Authority, which concluded that no ethical approval was required and that there was no risk of ethical dismay, as the study was not handling any sensitive information. However, the ethical guidelines from the Helsinki Declaration were followed throughout the study (World Medical Association, 2013). Hence, all participants were provided with both verbal and written information regarding the study and were informed that participation was voluntary and that their personal information would remain strictly confidential throughout the study.

Access to data was restricted solely to the researchers involved in this study, and the data were pseudonymized. The first and last authors also took proactive measures to establish a trustworthy and respectful environment for the participants. Oral and written informed consent, which outlined participants’ right to withdraw from the study at any point, was obtained from all participants prior to the focus group discussions.

## Results

**Figure 1.**
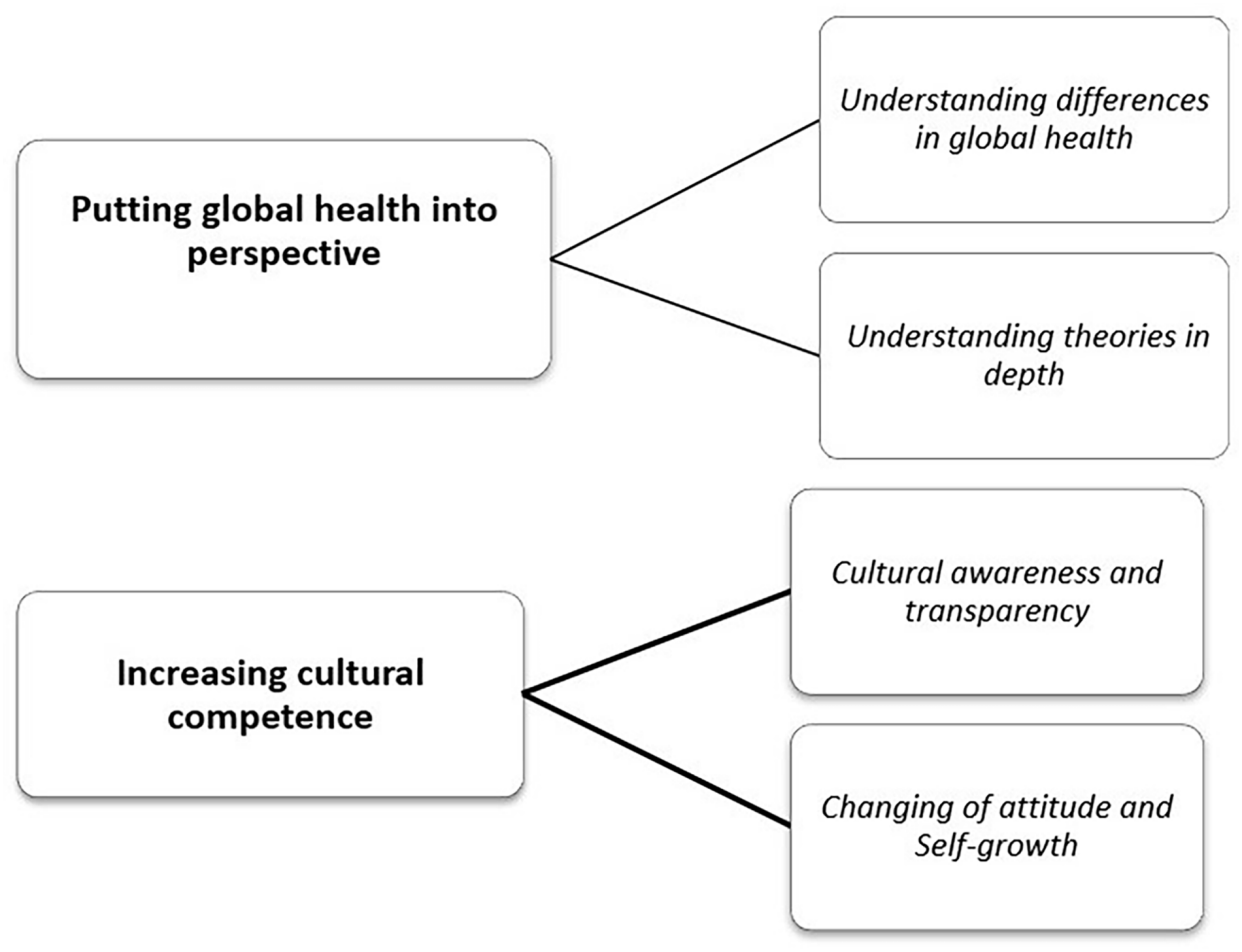
Identified Categories and Subcategories.

Two main categories were identified through the analysis: *putting global health into perspective* and *increasing intercultural competence*, each with two subcategories (see Figure 1).

### Putting Global Health Into Perspective

#### Understanding Differences in Global Health

Nursing students in both Sweden and Somaliland experienced their participation in the collaborative virtual seminars as educative. They valued the opportunity to connect and share professional ideas with students from both the Global North and Global South. They expressed that the most significant experience gained from the collaborative virtual seminars was sharing and learning about climate change and its impact on global health. Both the Somali and Swedish students indicated that due to the collaborative virtual seminars, they better understood that the role nurses play in sustaining the global climate is vital for global health care:

We have come a long way, but still quite a lot of work needs to be done with global climate change problems. I think through the collaborative virtual seminar, we, nursing students, can contribute by sharing experiences and building ideologies that promote global health. (ID, Student 4, DU)

The Somali nursing students expressed that they gained a better understanding of their differences from and similarities to Swedish nursing students regarding global climate hazards. They perceived that due to climate change, it was more common to experience noncommunicable diseases in the Global North than in the Global South, where communicable diseases were more common. They reflected on this knowledge as vital for improving and sustaining global health:

We have learned how different climate change problems can be managed. . . and how we can manage as nurses. Because as a future nurse, you are supposed to work globally and not only in your country. (FGD-1, Student 3, UoH)

The students stated that due to climate change, numerous individuals in particularly susceptible regions are vulnerable and face the potential inability to care for themselves. They reflected on the pivotal role of governmental support as a crucial resource for helping the vulnerable and maintaining their health. The Somali nursing students considered strategies used to mitigate climate change and how the Global North manages climate change. For example, they learned that in Sweden, not only is the government responsible for reducing climate change but individual citizens and the society as whole are also engaged in climate change debates and are proactive in preventing climate change and its hazards. The Somali nursing students stated that they found the knowledge gained from the collaborative virtual seminars empowering in relation to how they, as future nurses, can be involved in and work with climate change issues.

In contrast, the Swedish students expressed that climate change was more prominent in Somaliland than in Sweden. They reflected on the availability and high consumption of resources in the Global North, realizing that the effect of such consumption on climate change affects the Global South the most, thereby resulting in increased poverty, migration, and communicable diseases. The Swedish nursing students expressed gratitude for their own resources and became more mindful of the usage of these resources due to their participation in the collaborative virtual seminar. They also indicated that sharing with and learning from the Somali students gave them a sense of realization and reflection that led to other perceptions.

Swedish nursing students expressed that they had gained a clearer understanding that climate change is a global problem that manifests uniquely in specific countries:

I remember it was during summer, and it was so hot, and every one of us Swedish students had water bottles in our hands while they told us about their water shortage. You get a bad conscience when sitting there and with the advantage, but at the same time, you should not really be ashamed of it either. . . but we should be grateful for it and try to share what we have. (FGD-3, Student 2, DU)

#### Understanding Theories in Depth

All participants indicated that the structure of the collaborative virtual seminars created a means of putting theory into practice. Swedish nursing students expressed that the collaborative virtual seminars provided not only knowledge regarding global health but also an understanding of related facts and theories. One student explained that before the seminar, the nursing students were required to acquire theoretical knowledge from the literature on global health and to present and discuss it during the seminar. Through the students’ presentations and discussions on global health, they gained in-depth knowledge of how theories are applied to varied real-life contexts:

It was so good reading and getting theoretical knowledge before doing the twinning because then we could relate to and implement the theoretical knowledge in practice. (FGD-3, Student 3, DU)

Participants expressed satisfaction with holding professional discussions regarding global health with nursing students from another continent in a manner that felt collegial and like a partnership. They stated that the knowledge gained from the seminar would benefit their roles as future global nurses when encountering patients from different cultures and participating in global health promotion.

### Increasing Cultural Competence

#### Cultural Awareness and Transparency

All the participants indicated that the seminars broadened their cultural perspective, and most of them expressed that the seminar increased their cultural awareness (in terms of differences and similarities between the other culture and their own); however, a few were unsure whether this was due to their participation in the collaborative virtual seminars alone or earlier personal encounters, such as interactions with people from other cultural backgrounds. Moreover, the Swedish students stated that they acquired a sense of cultural transparency—or a “bigger picture of reality.”

From the topics discussed in the collaborative virtual seminars, the nursing students gained transparency regarding the influence of culture in relation to life situations, education, family dynamics, and individuals’ and society’s roles in the two countries. With this cultural transparency, the nursing students became better equipped to reflect on patients’ health needs through the lens of their cultural background and life experiences.

#### Changes in Attitude and Self-Growth

The nursing students indicated that because of the discussions held during the collaborative virtual seminars, their attitudes toward meeting patients from other cultural backgrounds changed. A Somali nursing student reflected on the knowledge acquired regarding the risks of discrimination arising from language barriers and biases toward other ethnicities. They also noted a shift in their attitudes toward encountering and caring for patients during a hospital internship. A Swedish nursing student expressed a shift in attitude and acknowledged the importance of not using one’s own cultural experiences as a benchmark when interacting with patients from diverse cultures, which can help prevent nurses from inadvertently neglecting a patient’s specific health needs that may be based on cultural aspects. Nursing students from both universities expressed that participating in the collaborative virtual seminars led to self-growth and made them more humble, open, and responsive to cultural differences and cultural needs in providing health care:

For me . . . it probably makes me more responsive, humble, responsible, and better at taking others into account, as well as their cultural background. This humility will help us when we start working as nurses, I hope. (ID, Student 4, Somali)

Furthermore, the participants stated that participating in the collaborative virtual seminars increased their cultural curiosity, thereby making them more interested in learning about other cultures, health care, and new perspectives. For certain students, engagement in the collaborative virtual seminars pushed them beyond their comfort zones and introduced them to a novel cultural experience. They also reflected on the impact of increased global migrations on health care and the importance of nurses acquiring cultural competence to avoid cultural insensitivity. They expressed deep appreciation for the opportunity to be involved with nursing students across continents. Moreover, all the participants indicated that they now understood that when caring for patients from diverse cultures, consideration of cultural differences and needs is necessary.

## Discussion

Cultural awareness is a contributing factor to developing cultural competence and sensitivity (Campinha-Bacote, 2018). Open-mindedness and cultural competence are crucial for nurses to provide meaningful, effective, and safe care for patients (McFarland & Wehbe-Alamah, 2018; Sanford et al., 2021; Sharifi et al., 2019). The findings of this study showed that by participating in the collaborative virtual seminars, nursing students gained an understanding of global health, cultural awareness, and cultural transparency, and experienced self-growth and changes in attitudes. The students expressed experiencing increased cultural curiosity and an awareness of their responsibility, as aspiring global nurses, to contribute to sustainable global health care.

Wihlborg et al. (2018) stated that collaborative virtual and transformative learning contributes to critical reasoning, recognition of differences and similarities, and an enhancement of students’ perspectives. Through their participation in the collaborative virtual seminars, students cultivated a drive to foster and uphold global health perspectives, which led to the development of cultural awareness and curiosity. We believe that such seminars can help address the call to engage nursing students in the advocacy for global health perspectives (Fields et al., 2021; UN, 2015; Upvall & Luzincourt, 2019; Wilson et al., 2016).

However, it is important to emphasize that in collaborative virtual seminars, the emphasis should not be on *eliminating* differences but rather on *understanding* both differences and similarities. It is also important to bear in mind that while collaborative virtual seminars can be used as a platform for equipping nursing students with the necessary knowledge to promote global health perspectives and cultural competence, they cannot replace the international student exchange practice.

Our findings revealed experiences of transformative learning as the benefits that the students expressed; they gained cultural awareness, curiosity, and a sense of change in attitude and self-growth. Although a few nursing students found it difficult to pinpoint whether and how their increased cultural competence was obtained from the collaborative virtual seminars. The findings highlight that university educators can use virtual seminars as a pedagogical tool to help nursing students develop knowledge and self-awareness, creating a base for lifelong learning and providing a forum for discussing and finding common ground.

### Limitations

Although this study contributes to understanding how collaborative virtual seminars between universities promote cultural competence and an understanding of global health, a few methodological limitations exist. One limitation is that the study focused only on students who participated in the collaborative virtual seminars and not on the entire class that undertook similar assignments. We did not compare whether students without exposure to the collaborative virtual seminar also demonstrated similar knowledge of global health.

Another limitation is the recall bias, as students were interviewed 3–6 months after the collaborative virtual seminars. Furthermore, conducting focus group discussions also has limitations. Participants knowing each other may result in similar perspectives and agreement among them. Although participants were explicitly instructed not to share the discussions held in the seminars outside the group, there was no absolute guarantee of confidentiality. However, the participants did refrain from discussing sensitive or personal information during the sessions. Moreover, since the students who participated in the collaborative virtual seminars can be assumed to be those with an interest in global issues, our findings cannot be generalized to all nursing students. However, our findings can be applied to similar settings.

## Conclusion

If nurses are to advocate for global health knowledge, future nurses must be involved in educational activities that promote global health and cultural competence. This study revealed that promoting global health knowledge and cultural competence through collaborative virtual seminars among nursing students is beneficial, as it generates awareness and actualization of topics, such as global health and cultural competence.

Collaborative virtual seminars can also be used as a platform for global nursing, building a global community with a common understanding of global health, and raising awareness among future nurses of patients’ safety and cultural competemility in health care. Such global institutional collaboration benefits universities because it fosters international collaboration not only between nursing students but also between educators and researchers.

However, more can be done to conduct seminars between countries that are socially, economically, and culturally distant from each other. When planning the nursing curriculum and related activities, educators should focus on critical thinking, awareness, actualization of the topic, and students’ involvement. These are important areas for future research into how sustaining virtual collaborative international activities can promote nurses’ global health and cultural competence.
